# Pediatric COPA Syndrome Overlapping With Heterozygous Familial Mediterranean Fever: A Dual Inflammatory Disorder

**DOI:** 10.1155/crpe/9123193

**Published:** 2025-10-19

**Authors:** Laith Khalaf, Meera Lahlouh, Kareem Abdul-Haleem, Mohammad Hamdan

**Affiliations:** ^1^Medicine & Allied Medical Sciences Faculty, Department of Medicine, An-Najah National University, Nablus, State of Palestine; ^2^Pediatric Allergy and Immunology, Jenin Governmental Hospital, Jenin, State of Palestine

## Abstract

We present the case of a 6-year-old Palestinian girl who suffered from recurrent attacks of arthralgia, abdominal pain, fever, and mesenteric lymphadenopathy over the past 3 years. Despite the presence of all clinical diagnostic criteria for Familial Mediterranean Fever (FMF) and a heterozygous mutation (p.V726A) in the Mediterranean Fever (MEFV) gene, the atypical presentation of these symptoms prompted a comprehensive genetic examination. This revealed COPA syndrome as an additional diagnosis, a rare autosomal dominant disorder of immune dysregulation that affects the lungs, joints, and occasionally the kidneys. Following colchicine therapy, patient's symptoms and lymphadenopathy decreased, indicating a significant recovery. We emphasize the importance of a comprehensive genetic examination in children with complex symptoms and doubt about the diagnosis to enable early detection of rare disorders and prompt initiation of treatment.

## 1. Introduction

Familial Mediterranean Fever (FMF) is a monogenic autoinflammatory disease that affects people of Mediterranean origin, including Palestinians. It is characterized by recurrent attacks of arthritis, fever, and serositis [1]. FMF is inherited in an autosomal recessive pattern and results from pathogenic mutations in the Mediterranean Fever (MEFV) gene, particularly in exon 10. The M694V mutation is the most common mutation associated with the disease [2]. Although the disease usually occurs in homozygous carriers, recent evidence suggests that 4%–15% of heterozygous carriers develop FMF-like symptoms, particularly with severe mutations, such as p.V726A [3, 4].

In a 10-year-long study on Palestinian children, 14% of heterozygous MEFV mutation carriers improved upon treatment with colchicine, particularly if they had a family history of FMF or associated complications such as amyloidosis [4, 5]. Current studies on inflammatory markers have shown that numerous individuals with a heterozygous mutation experience milder symptoms, but many of them improve when they take colchicine [3, 6]. This suggests that there is variation in symptom severity even among carriers of a heterozygous mutation.

A whole-exome sequencing revealed that our patient also had the coatomer subunit α (COPA) syndrome. This syndrome was first described in 2015, and it is caused by an autosomal dominant mutation in COPA gene that controls transport within cells [7]. COPA differs from FMF because it affects both innate and acquired immunity, leading to long-term inflammation that can affect the lungs and kidneys [7]. Studies have shown that symptoms begin to manifest between the ages of 3 and 5, and they consist of anemia, which is hard to cure, low natural killer (NK) cell numbers, and high Antinuclear Antibody (ANA) levels [7].

Approximately 40 families have been documented to have COPA syndrome worldwide, with an incidence rate of approximately 1 per million; however, diagnosis is increasing due to increased genetic testing capabilities [7]. In this case, we focus on the diagnostic complexity in children with symptoms ranging from immune dysregulation to autoinflammatory disorders. We emphasize the importance of a comprehensive genetic evaluation in some cases, even in the presence of a preliminary genetic diagnosis that may explain the clinical symptoms [3–5].

## 2. Case Presentation

A 6-year-old Palestinian female, weighing 23 kg (50^th^ percentile), born to nonconsanguineous parents, has been suffering from recurrent unexplained episodes of fever up to 39°C, accompanied by joint pain, vomiting, and abdominal pain for the last 3 years. Her symptoms were occurring every 10 days and lasting for 1-2 days, then resolving spontaneously. She had no history of rash, oral ulcer, or chest pain. Examination of the patient's joints revealed no swelling or redness, and she had a normal range of motion.

Due to the persistence of her symptoms, she was evaluated multiple times in both Gastroenterology and Rheumatology clinics. Upon examination, she is performing well academically and has no distinctive facial features, organomegaly, abnormal skin lesions, or neuromuscular abnormalities. Her height was 129 cm (95^th^ percentile), and her head circumference was 51 cm, consistent with normal growth. Several ultrasounds were performed, and mesenteric lymphadenopathy was consistently found. Investigations for stool occult blood and *Helicobacter pylori* were negative; despite that, she had normocytic anemia (hemoglobin: 9.7 g/dL; MCV: 81 fL), and clinical improvement was noted after taking iron supplements (hemoglobin: 11.6 g/dL; MCV: 84.2 fL). Both upper and lower endoscopies showed normal mucosa, and biopsies from the duodenum and terminal ileum were unremarkable. Rheumatoid factor (RF) and ANA profile tests returned negative. A complete blood count (CBC) showed no abnormalities, and liver function tests were within the normal range. Inflammatory markers, including C-reactive protein (CRP) and Erythrocyte Sedimentation Rate (ESR), were all within the normal limits.

Following these findings, she was started on colchicine 0.5 mg once daily for 2 months, and genetic testing was ordered after the patient was referred to a genetic counsellor. Due to the persistence of her arthralgia and abdominal pain, the dose was increased to twice daily. This was followed by clinical improvement as she stopped having fever, and her joint and abdominal pain decreased, as well as lymphadenopathy, and she experienced no recurrences during the 2 months of follow-up. After improvement, her colchicine dose was tapered to 1.5 tablets daily. Her whole-exome sequencing identified that she is a carrier of a heterozygous pathogenic variant in the MEFV gene (p.V726A). After that, the patient was referred for further evaluation, including a flow cytometry analysis, which demonstrated normal T-cell and B-cell counts, a normal CD4:CD8 ratio, but decreased NK cell counts.

Following these findings, she was referred to a genetic center for another whole-exome sequencing to evaluate for another genetic disorder, which revealed that she had a heterozygous missense variant in the COPA gene (p.T595A) at exon 18, indicating a concurrent COPA syndrome. After detecting this syndrome, we tested her creatinine level, which was 0.35 mg/dL. Respiratory-wise, the patient was asymptomatic, with a normal oxygen saturation above 94% on room air, equal air entry bilaterally, no wheezes or added sounds, and never complained of cough or dyspnea; as a result, no further testing was performed. Therapy was maintained on 1.5 tablets of 0.5 mg colchicine daily.

Genomic amplification of exon 18 of the COPA gene (NM_004371.4) and direct sequencing were performed to detect the presence of c.1783A > G, pT595A variant of the DNA extracted from peripheral blood samples for the patient's parents. We found that the father is heterozygous for this variant, while the mother is normal. No further investigations were done for the father as he was asymptomatic.

## 3. Discussion

We present a case of a 6-year-old female with recurrent episodes of fever, mesenteric lymphadenopathy, arthritis, and abdominal pain since the age of 3 years. She was suspected of having FMF based on her typical presentation that fulfills the Tel Hashomer Criteria, which requires at least two major symptoms or one major symptom with one minor symptom to diagnose FMF. Major symptoms include recurrent febrile episodes with serositis, a favorable response to colchicine, and amyloid A (AA) amyloidosis, while minor symptoms include recurrent febrile episodes, FMF in a first-degree relative, and an erysipelas-like erythema. Our patient fulfilled the criteria by having recurrent febrile episodes with abdominal pain and a good response to colchicine therapy [8]. Genetic testing revealed a heterozygous V726A mutation in the MEFV gene. However, the presence of other unexplained symptoms, like her abnormal flow cytometry and lymphadenopathy, raised our suspicion of another genetic disease, so we ordered another whole-exome sequence, which confirmed the diagnosis of COPA syndrome. Upon diagnosis of FMF, she started on colchicine with a good clinical response.

FMF is the most common autoinflammatory disease worldwide, mostly affecting people from the Eastern Mediterranean region, with Turkey having the highest number of reported cases [9]. It is caused by an autosomal recessive gain-of-function mutation in the MEFV gene located on the short arm of chromosome 16 (16p13.3), which encodes the pyrin protein. This protein regulates the innate immune system by controlling the activation of IL-1β and IL-18 via caspase-1. As a result, overactivation of pyrin leads to frequent inflammatory attacks that cause the major symptoms of FMF [9]. Many variants were identified for the MEFV gene mutations, with M694V being the most common, followed by M694I, V726A, and M680I. These four variants account for almost 75% of all FMF patients [2, 9].

Typically, FMF patients present with spontaneously resolving episodes of fever, peritonitis, pleuritis, arthritis, and erysipelas-like erythema, with each episode lasting 1-3 days [9]. Many diagnostic criteria were proposed, with the Tel Hashomer Criteria being the most commonly used and most sensitive, requiring at least 1 major or 2 minor criteria to diagnose [8, 9]. Genetic testing is not required for diagnosis, as FMF is a clinical diagnosis, but it is the only confirmatory test.

As an autoinflammatory disease, FMF can lead to the accumulation of acute-phase reactants like serum amyloid-associated protein, leading to AA amyloidosis, which is the most common and serious complication [8, 9]. However, FMF is a highly treatable disease once diagnosed, with colchicine being its mainstay treatment, having a good prognosis, but its narrow therapeutic index requires regular monitoring [5, 8, 9].

In contrast, COPA syndrome is a rare genetic disease that was first reported in 2015 as an autoinflammatory disease. Further studies revealed that it is caused by a missense autosomal dominant mutation that affects both innate and adaptive immune systems, so it is more accurate to classify it as an immune dysregulation disorder [10, 11]. The most commonly affected organs are the lungs, manifesting as diffuse alveolar hemorrhage (DAH) and/or interstitial lung disease (ILD), followed by joints, which manifest as nonerosive arthritis. Although less common, renal involvement was mentioned and may present as different types of glomerulonephritis [7, 10, 11].

Affected infants often have failure to thrive, nonspecific lethargy, and unexplained anemia before diagnosis, with 3.5 years being the average age at presentation [10]. Anemia is usually normocytic with low iron levels and high reticulocyte count, mimicking hemolytic anemia, and is thought to be caused by insidious alveolar hemorrhage in these infants [10, 11]. Laboratory findings in COPA syndrome are usually nonspecific and can mimic other autoimmune and autoinflammatory diseases. Positive ANA, RF, and anticyclic citrullinated antibodies are commonly detected, with elevated inflammatory markers (ESR and CRP). Treatment still needs further studies, but immunosuppressive therapy is required, and some patients may require bilateral lung transplantation [10, 11].

Genetic testing of our patient revealed that she has a heterozygous MEFV gene mutation despite having a typical FMF presentation. Studies have shown that up to 30% of FMF patients have heterozygous MEFV gene mutations, but these patients have milder symptoms, unlike our case. Furthermore, exome sequencing confirmed the presence of COPA syndrome despite the absence of typical markers like RF and ANA profile, and normal CRP and ESR.

The patient had a history of normocytic anemia (Hb = 9.7 g/dL; MCV = 81 fL) and was treated with iron supplementation. The cause of her anemia was not determined, but it may be caused by COPA syndrome, so further evaluation is needed. She did not have another episode of anemia after initiating colchicine; however, her inflammatory markers were normal before therapy, excluding anemia of chronic disease. Additionally, immunosuppressive therapy should be started, and extensive pulmonary assessments should be done to screen for DAH and ILD.

Due to the coexistence of two genetic disorders, we explained to her family that they are at higher risk of having these diseases, especially her younger sister, who started having similar symptoms of abdominal pain and fever. Exome sequencing was recommended for all family members. We also advised delaying any future pregnancies until all genetic testing is completed. We also advised the mother to undergo fetal genetic counseling for all her future pregnancies. The mutation was detected in her father only, but he was asymptomatic, so no further investigations were done.

## 4. Conclusion

The difficulties physicians face when diagnosing atypical recurrent inflammatory syndromes in children, such as in our case, are evident. Given the patient's initial symptoms and her response to colchicine, a diagnosis of FMF was made, particularly after detecting the MEFV p.V726A mutation. There was no known explanation for other concerning symptoms, like her normocytic anemia, which was treated with iron therapy without identifying its cause. A comprehensive assessment of these symptoms would have allowed for the early detection of COPA syndrome, a rare syndrome discovered through subsequent genetic testing in this case. We highlight the risks of relying on a single diagnosis in complex cases with atypical symptoms. We emphasize the need to seek additional diagnoses in complex cases and to perform comprehensive genetic testing when necessary to enable early initiation of treatment.

## References

[1] Lidar M., Livneh A. (2007). Familial Mediterranean Fever: Clinical, Molecular and Management Advancements. *The Netherlands Journal of Medicine*.

[2] Shahbaznejad L., Raeeskarami S. R., Assari R. (2018). Familial Mediterranean Gene (MEFV) Mutation in Parents of Children WITH Familial Mediterranean Fever: What Are the Exceptions?. *International Journal of Inflammation*.

[3] Elhani I., Backes S., Kallinich T. (2024). Inflammatory Biomarker Analysis Confirms Reduced Disease Severity in Heterozygous Patients With Familial Mediterranean Fever. *RMD Open*.

[4] Shrateh O. N., Thalji M., Jobran A. W. M., Brakat A. M., Attia A. M., Abunejma F. M. (2023). Genotype Mutations in Palestinian Children with Familial Mediterranean Fever: Clinical Profile, and Response to Colchicine Treatment: A Retrospective Cohort Study. *Mediterranean Journal of Rheumatology*.

[5] Çakan M., Alkaya A., Koru L. (2024). The Journey of MEFV Heterozygous Children: With or Without Colchicine. *European Journal of Pediatrics*.

[6] Cam V., Unal D., Sag E. (2025). Re-Evaluation of MEFV Carriers Previously Diagnosed With Familial Mediterranean Fever: A Colchicine Discontinuation Study. *Rheumatology*.

[7] Watkin L. B., Jessen B., Wiszniewski W. (2015). COPA Mutations Impair ER-Golgi Transport and Cause Hereditary Autoimmune-Mediated Lung Disease and Arthritis. *Nature Genetics*.

[8] Bashardoust B. (2015). Familial Mediterranean Fever; Diagnosis, Treatment, and Complications. *Journal of Nephropharmacology*.

[9] Tufan A., Lachmann H. J. (2020). Familial Mediterranean Fever, From Pathogenesis to Treatment: A Contemporary Review. *Turkish Journal of Medical Sciences*.

[10] Padureanu V., Forțofoiu M. C., Donoiu I. (2024). COPA Syndrome—From Pathogenesis to Treatment. *Diagnostics*.

[11] Simchoni N., Vogel T. P., Shum A. K. (2023). COPA Syndrome from Diagnosis to Treatment: A Clinician’s Guide. *Rheumatic Disease Clinics of North America*.

